# Overexpression of Pim-1 in bladder cancer

**DOI:** 10.1186/1756-9966-29-161

**Published:** 2010-12-11

**Authors:** Shengjie Guo, Xiaopeng Mao, Junxing Chen, Bin Huang, Chu Jin, Zhenbo Xu, Shaopeng Qiu

**Affiliations:** 1 Department of Urology, the First Affiliated Hospital, Sun Yat-Sen University, Guangzhou, 510080 China; 2 School of Food Science and Nutrition, Leeds University, Leeds LS2 9JT, UK; 3 Colleges of Light Industry and Food Sciences, South China University of Technology, Guangzhou, China; 4 Department of Microbial Pathogenesis, Dental School, University of Maryland, Baltimore, MD-21201, USA

## Abstract

**Background:**

Pim-1 is a serine-threonine kinase which promotes early transformation, cell proliferation and cell survival during tumorigenesis. Several studies have demonstrated that Pim-1 kinase play a role in different cancer types, however, the function of Pim-1 in bladder cancer is poorly understood.

**Methods:**

Expression and localization of Pim-1 in human normal and malignant bladder specimens were examined by Immunohistochemistry and Pim-1 staining score was compared with several clinicopathologic parameters. To further demonstrate the biological function of Pim-1 in bladder cancer, its expression was validated in five bladder cancer cell lines by western blot and immunohistochemistry analyses. Subsequent knockdown of Pim-1 was achieved by lentivirus encoding small interfering RNA, and the effect of Pim-1 on bladder cell survival and drug sensitivity were further assessed by colony formation and cell proliferation assays.

**Results:**

When compared with normal epithelium, Pim-1 was overexpressed in bladder cancer epithelium, and the expression level was higher in invasive bladder cancer than Non-invasive bladder cancer specimens. Pim-1 was also detected in all the bladder cancer cell lines examined in our study. Moreover, the knockdown of Pim-1 significantly inhibited bladder cancer cell growth and also sensitized cells to chemotherapeutic drugs *in vitro*.

**Conclusions:**

Our results in this study suggest that Pim-1 may play a role in bladder cancer initiation and progression. Since Pim-1 is also involved in bladder cancer cell survival and drug resistance, Pim-1 is a potential candidate for targeted therapy in bladder cancer.

## Background

Bladder cancer is one of the most common types of cancer globally, with approximately 75% of the diagnosed tumors classified as Non-invasive tumor (Ta, Tis, or T1). Treatment of Non-invasive tumor includes transurethral resection (TUR) with or without intravesical instillation therapy, but the recurrence rate is high, ranging from 50% to 70%. In addition, an average of 10% to 20% for Non-invasive tumors may further progress to muscle-invasive disease, thus lead to eventual radical Cystectomy and urinary diversion [1-3]. In this context, clinicians face challenges to identify the novel therapeutic targets for bladder cancer.

Pim-1 is overexpressed in several types of cancer, including lymphoid and haematopoietic malignancies [4], prostate cancer [5], squamous cell carcinomas [6], gastric carcinoma and colorectal carcinomas [7]. Currently available studies have demonstrated that the expression of Pim-1 can be predictive of tumor outcome following chemotherapy and surgery, and it is correlated with the enhanced metastatic potential of the tumor[8]. As a member of serine/threonine kinase family, Pim-1 has multiple roles in tumorigenesis such as promoting transformation and cell proliferation partly through regulation of cell cycle and transcription by phosphorylating of number of substrates including cdc25A/C, HP1, and p100 [9-11]. Moreover, it has been shown that Pim-1 may play a role in the regulation of the survival signaling through the modulation of Bcl-2 family member including Bad, Bcl-2 and Bcl-XL [12-14]. However, the expression and significance of Pim-1 in bladder cancer remains unknown. Therefore, the aims of the present study are to investigate the expression level of Pim-1 in bladder cancer tissue and study its function in the pathogenesis and progression of bladder cancer.

## Methods

### Patient samples

Sixty-six clinical bladder samples isolated from the First Affiliated Hospital of the Sun Yat-Sen University (Guangzhou, China), were examined in the present study. All patients including forty-eight men (72.3%) and eighteen women (27.7%), had been treated for urothelial carcinoma of the bladder by transurethral resection of bladder (TUR) or Cystectomy and were diagnosed with a bladder cancer for the first time at an average age of 56 years (range, 33-78 years). Pathologic staging and grading were performed according to the 2002 TNM classification system and World Health Organization criteria, respectively. The use of the human tissue in this study was approved by the Ethics Council of the Sun Yat-Sen University for Approval of Research Involving Human Subjects.

### Immunohistochemistry

All 5μm thick paraffin sections were deparaffinized with xylene and rehydrated through graded alcohol washes, followed by antigen retrieval by heating sections in sodium citrate buffer (10 mmol/L, pH6.0) for 30 minutes. Endogenous peroxidase activity was blocked with 30 min incubation in 0.03% H_2_O_2 _in methanol. The slides were then blocked by incubation in normal goat serum (dilution 1:10) in PBS (pH 7.4) and subsequently incubated for monoclonal mouse IgG1 anti-Pim-1 antibody(sc-13513; Santa Cruz Biotechnology, Santa Cruz, CA, USA) with 1:30 dilution at 4°C overnight. Following this step, slides were treated with biotin-labeled anti-IgG and incubated with preformed avidin-biotin peroxidase complex. Control staining of the same sections was performed with the preimmune primary antibody, and no Pim-1 immunostaining was observed in these sections. The sections were briefly counter-stained with hematoxylin. IHC reactions for all samples were repeated at least three times, and typical results were illustrated.

### Scoring and Statistical analyses

The staining of Pim-1 was graded in each sample based on the intensity of the immunoreactivity in the cancer cells and was stratified as strong staining (3), moderate staining (2), weak staining (1) and negative (0). Using these criteria, the immunostaining results were evaluated independently by XPM and BH. The correlation of interobserver was calculated from the independent evaluations. For cases with discrepancy, a consensus was reached during a common evaluation session. The statistical analyses were carried out by using SAS version 9.0 statistics software (SAS Institute, Inc., Cary, NC).

### Cell culture and lentiviral infection

Bladder cancer cell lines T24, UM-UC-3, 5637, J82 and RT-4 were purchased from the American Type Culture Collection. UM-UC-3 and T24 cells were grown in Dulbecco's modified Eagle's medium. 5637, J82 and RT-4 cells were maintained in RPMI 1640 with 10% fetal bovine serum and 1% (v/v) penicillin and streptomycin (100 μg/ml) and maintained at 37°C in a 5% CO_2 _atmosphere. The infection of lentivirus of Pim-1 siRNA was carried out as reported previously [15].

### Western Blot

Western blot was performed as described previously [16]. Briefly, the equal amounts of sample were resolved on a SDS polyacrylamide gel and transferred to a polyvinylidene difluoride membrane. Blots were incubated with the indicated primary antibodies overnight at 4°C and followed by detection with horseradish peroxidase-conjugated secondary antibody. The monoclonal anti-Pim-1 antibody was used at the dilution of 1:300, whereas anti-tubulin, Bcl-2, Bad and p-Bad (Ser112) (Santa Cruz Biotechnology, Santa Cruz, CA, USA) were used at the dilution of 1:2,000.

### Cell immunoperoxidase staining

Bladder cancer cells were plated onto the glass slides. After 24 h, cells were fixed with ice-cold acetone. The endogenous peroxides activity was inactivated by incubating cells with 0.03% H_2_O_2 _for 10 min. Slides were then incubated with Pim-1 antibody at room temperature for 1 hour and followed by horseradish peroxides-conjugated anti-mouse Ig (Chemicon; 1:500 dilutions). Finally, slides were incubated with biotin-labeled anti-IgG avidin-biotin peroxidase complex and developed with DAB Solution.

### Colony formation assay

The cells (1 × 10^4^) were seeded in 6-well plate and infected with the lentivirus expressing control siRNA or Pim-1 siRNA. Cell culture was maintained in complete medium for two weeks. The cell colonies were then visualized by Coomassie blue staining.

### Drug-sensitivity assay

Cells were infected with lentivirus encoding control siRNA or Pim-1 siRNA. At 48 h post-infection, cells were seeded on 96-well plate at a density of 6 × 10^3 ^cells/well. After 24 h, cells were treated with various doses of Doxorubicin or Docetaxel (Sigma, St Louis, MO, USA) for another 48 h. The cells viability was measured by the WST-1 (Roche) assay following the manufacturer's instructions.

## Results

### Overexpression of Pim-1 in human bladder cancer specimens

**Figure 1:**
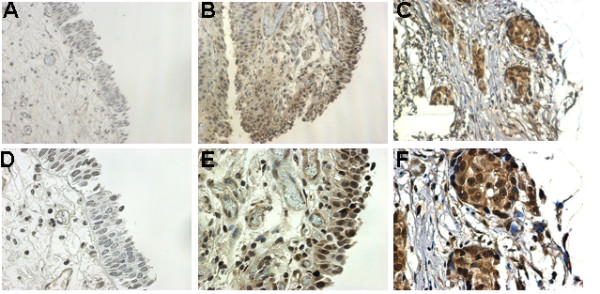
**Overexpression of Pim-1 in human bladder cancer specimens**. Pim-1 is overexpressed in both cytoplasm and nucleus of bladder cancer cells. Normal bladder epithelium cells show no or minimal staining (A&D). Bladder cancer cells show cytoplasm and nucleus positive staining (B&E). Invasive bladder cancer cells show strong staining(C&F). Magnification × 200 (A, B, C), or × 400 (D, E, F).

To validate the expression of Pim-1 protein in bladder cancer, human bladder specimens containing normal epithelium (n = 21) and malignant tissues (n = 45) were studied by immunohistochemistry using Pim-1 antibody. The staining data showed that Pim-1 expression is weakely detect in the epithelial cells of normal bladder epithelium, however, most of the malignant bladder epithelial cells exhibited Pim-1 immunoreactivity in both cytoplasm and nuclear (Figure 1). For further analysis, the immunoreactivity of Pim-1 was divided into negative (score 0-1) vs. positive (score 2-3) subgroups. Detailed staining scores in normal and malignant bladder specimens are presented in Table 1, which showed that Pim-1 expression is significantly higher in bladder cancer specimens (84.4%) than in normal specimens (9.5%) (p < 0.001), suggesting an overexpression of Pim-1 at the translational level in bladder cancer.

**Table 1:** Pim-1 immunostaining intensity in human normal and maligancy bladder tissues

groups	n	negtive	positive
Normal	21	19(90.5%)	2(9.5%)
Malignancy	45	7(15.6%)	38(84.4%)

*p *< 0.001

To explore potential correlations between the expression of Pim-1 and tumor progression, malignant bladder specimens were further classified into Non-invasive (Tis, Ta and T1) and invasive (≥T2) groups. The data (Table 2) shows that the staining intensity of Pim-1 is increased in invasive bladder carcinoma samples (95%) when compared with Non-invasive bladder cancer specimens (76%)(p < 0.01). However, correlation of Pim-1 within different tumor grades was not observed (data not shown). Taken together, Pim-1 may be associated with bladder cancer initiation and progression.

**Table 2:** Pim-1 immunostaining intensity in No-invasive and Invasive bladder tumors

groups	n	negtive	positive
Non-invasive	25	6(24.0%)	19(76.0%)
Invasive	20	1(5%)	19(95.0%)

*p *< 0.01

### Expression profile of Pim-1 in bladder cancer cell lines

**Figure 2:**
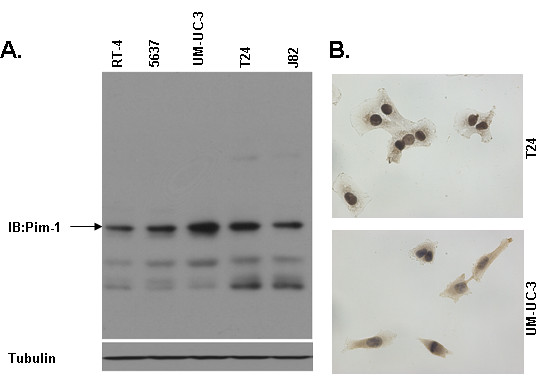
**Expression profile of Pim-1 in bladder cancer cell lines**. A. Expression profile of Pim-1 in bladder cancer cell lines. Cell lysate from five bladder cancer cell lines were examined by western blot for Pim-1. Tubulin is as the loading control. B. The expression and localization of Pim-1 in human bladder cancer cell lines. Cells were immunoperoxidase stained with Pim-1 antibody as described as methods. Original magnification ×400.

In order to further demonstrate the role and function of Pim-1 in bladder cancer, the expression level of Pim-1 was validated in bladder cancer cell lines using western blot. As shown in Figure 2A, Pim-1 is expressed in all five bladder cancer cell lines at variable levels, with the maximum level in highly invasive cancer cell lines T24 and UM-UC-3.

The localization of Pim-1 in bladder cancer cells was confirmed by immunoperoxidase staining and as the results showed that Pim-1 was detected in all human bladder cell lines examined, including T24, UM-UC-3, 5637, J82 and RT-4. Representative images are presented in Figure 2B. The positive signals were primarily immunolocalized in both cell cytoplasm and nucleus, while some cell membrane staining is also detected.

### Pim-1 is essential for bladder cancer cell survival

**Figure 3:**
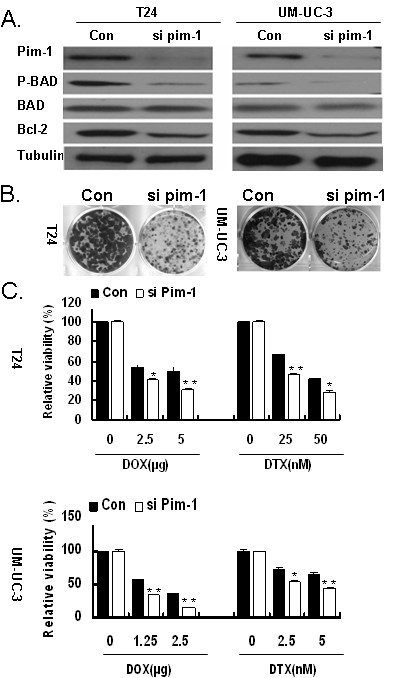
**Downregulation of Pim-1 inhibited the bladder cells growth and sensitized them to Doxorubicin and Docetaxel treatment**. A. Knockdown of Pim-1 decreased the phosphorylation of Bad and the expression of Bcl-2. The cells were infected lentivirus siRNA specific for Pim-1(si Pim-1) or vector control. At 48 h postinfection, cells were lysed and the lysates were subjected to western blot with indicated antibody. B. Downregulation of Pim-1 inhibited the bladder cancer cell growth. Total of 1 × 10^4 ^T24 and UM-UC-3 cells were plated in each well of a 6-well plate and infected with lentivirus encoding Pim-1 siRNA or vector control siRNA. The cell culture was maintained in complete medium for two weeks. Finally, the cell colonies were visualized by Coomassie blue staining. C. Decreased expression of Pim-1 sensitized bladder cancer cells to Doxorubicin and Docetaxel treatment. The cells were plated on 96 wells and infected with lentivirus encoding Pim-1 siRNA or vector control siRNA. At postinfection for 48 h, cells were treated with DOX (T24, 2.5 and 5μg/ml; UM-UC-3, 1.25 and 2.5 μg/ml) and DTX (T24, 25 and 50 nm; UM-UC-3, 2.5 and 5 nm) for another 48 h. The cell viability was assessed by WST-1 assay.*, p < 0.05 compared with the control; **, p < 0.01 compared with control.

To examine the biological significance of Pim-1, targeted knockdown of Pim-1 was achieved by lentivirus encoding siRNA specific for Pim-1 in T24 and UM-UC-3 cells, which express relatively high levels of Pim-1. The Pim-1 siRNA using in our experiments has been previously shown to specific knockdown Pim-1 in multiple prostate cancer cell lines [17,18]. As shown in Figure 3A, downregulation of Pim-1 decreased Phospho-Bad and Bcl-2 levels that are known to be regulated by Pim-1. Furthermore, downregulation of Pim-1 could also inhibit the cell growth and proliferation *in vitro *(Figure 3B), suggesting that Pim-1 may be important for the growth and survival of bladder cancer cells.

### Knockdown of Pim-1 sensitizes bladder cancer cells to chemotherapy *in vitro*

As Pim-1 is involved in drug resistance in some cancer types and adjuvant intravesical chemotherapy is one of the most common treatments in bladder cancer, we tested whether Pim-1 is also involved in drug response of bladder cancer cells. T24 and UM-UC-3 cells were treated with lentivirus encoding the siRNA specific for vector control or Pim-1 and then were tested for their responses to chemotherapeutic drugs. As shown in Figure 3C, downregulation of Pim-1 sensitized T24 and UM-UC-3 cells to Doxorubicin (DOX) and Docetaxel (DTX) when compared to the vector control. Our data implied that Pim-1 may contribute to the resistance of apoptosis and survival of bladder cancer cells in response to cytotoxic drugs.

## Discussion

In the present study we demonstrated for the first time that, Pim-1 was increased in human bladder cancer epithelium as compared with that in normal bladder tissue. When the tumors were stratified by Non-invasive and invasive, a statistically significant increase of Pim-1 expression was found in the subgroup of invasive tumor when compared with that in the Non-invasive tumor. Pim-1 was also detected in all human bladder cancer cell lines tested in our study. Knockdown Pim-1 led to decreased phosphorylation of Bad and reduced expression of Bcl-2. Furthermore, downregulation of Pim-1 inhibited the bladder cancer cells growth and sensitized them to chemotherapy *in vitro*. Further evaluation of the prognostic significance of Pim-1 in a larger cohort with sufficient follow-up times will allow better understand of the clinical significance of Pim-1.

Overexpression of the Pim-1 protein has been reported in hematolymphoid malignancies and solid cancers [4,5]. Pim-1 has been asserted to promote tumorigenesis through multiple mechanisms, including its interaction with other proteins such as c-myc, p27^KIP1^, p21^Cip1/WAF1^, Bad, Cdc25A/C dual specificity phosphates, androgen receptors and its ability to induce genomic instability [19-22]. The oncogenic effect of Pim-1 on non-haematopoietic malignancies is currently under investigation. Ellwood-Yen *et al *demonstrated that the overexpression of Pim-1, in cooperation with increased levels of c-myc, could lead to murine prostatic intraepithelial neoplasia and invasive adenocarcinoma in c-myc transgenic mice [23]. Taking into account the biological role of Pim-1 as an oncoprotein involved in cell cycle regulation and proliferative processes, our results suggested possible implication of Pim-1 in the initiation of bladder carcinogenesis. Moreover, upregulation of Pim-1 in invasive bladder cancer compared with Non-invasive tumors indicated that Pim-1 also may also contribute to bladder cancer progression.

Pim-1 has been considered as a survival kinase. Inhibition of Pim-1 results in a significant growth repression of prostate cancer cell [24]. Several inhibitors of Pim-1 have been shown to inhibit the growth of cancer cells, such as leukemic cells as well as prostate cancer cells. There are clinical trials to explore the safety of one of the Pim-1 inhibitor, SGI-1776, for the treatment of refractory non-Hodgkin's lymphoma and prostate cancer [25,26]. It also has been demonstrated that Pim-1 monoclonal antibody (mAb) could induce apoptosis in cancers cells of the prostate, breast and colon. Furthermore, the inhibition of Pim-1 function by treatment with Pim-1 siRNA, Pim-1 inhibitors or Pim-1 mAb sensitizes cancer cells to chemotherapy [15,27-29]. It is noteworthy that Pim-1 interacted and phosphorylated Bad, Etk and BCRP leading to antagonism of drug-induced apoptosis [14,17,18]. In bladder cancer, after an initial transurethral resection of bladder tumor (TURBT), adjuvant intravesical therapy is another treatment strategy used to reduce the risk of recurrence. However, the cancer recurrence rate is still high and the recurring cancer cells can become more resistant to further intravesical chemotherapy. It is necessary to identify an effective strategy to counter act challenges associated with clinical management of bladder cancer patients. In this regard, Pim-1 might be one of the potential therapeutic targets for the treatment of bladder cancer and further studies examining Pim-1 as a target of therapeutics are worthy of investigation.

## Conclusions

To the best of our knowledge, this is the first report showing overexpression of Pim-1 in bladder cancer and its association with bladder cancer cell survival, drug resistance and tumor progression. The current study offers significant information on the role and functions of Pim-1 in bladder cancer, and may aid in the development of novel therapy.

## Competing interests

The authors declare that they have no competing interests.

## Authors' contributions

XPM and BH evaluated the immunostainings. JXC and ZBX performed the statistical analysis. SJG and SPQ drafted the manuscript. JC revised the manuscript. All authors read and approved the final manuscript.
